# Ovarian cystadenofibroma: A masquerader of malignancy

**DOI:** 10.4103/0971-3026.73538

**Published:** 2010-11

**Authors:** Ashish Wasnik, Khaled Elsayes

**Affiliations:** Department of Radiology, University of Michigan Health System, Ann Arbor, MI - 481 05, USA

**Keywords:** Magnetic resonance imaging, ovarian cystadenofibroma, sonography

## Abstract

Ovarian cystadenofibroma is a relatively rare benign ovarian tumor that contains both epithelial and fibrous stromal components. The appearance of cystadenofibroma on imaging is often complex; cystic- to solid-appearing masses may be visualized and it often resembles a malignant tumor. Owing to the fibrous component of this tumor, MRI shows low-signal intensity on T2W images. This finding may help a radiologist make a preoperative diagnosis of this tumor and thus perhaps avoid aggressive surgical management.

## Introduction

Ovarian cystadenofibroma is a relatively rare benign tumor that is seen in women aged 15–65 years.[1] The routine imaging features of this tumor may mimic a malignant neoplasm, but the presence of the fibrous component often gives a specific/characteristic MRI appearance that may help differentiate it from malignant ovarian tumors.[2–5]

## Case Report

A 49-year-old female (gravida 1, para 1) presented to a gynecologist with complaints of right lower quadrant discomfort for 3–4 weeks, which was associated with nausea and poor appetite. There was no history of menstrual irregularity. Her last menstrual period was 4 weeks ago. An initial urine pregnancy test was negative. Physical examination revealed a normal uterus with a soft, mobile right adnexal mass.

**Figure 1:**
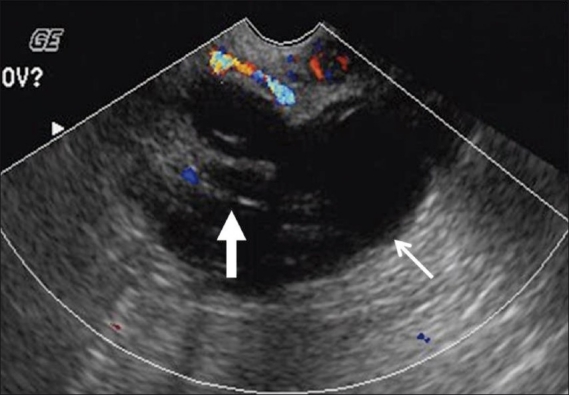
Transvaginal pelvic USG with color Doppler shows a complex cystic mass (thin arrow) with septae and solid components (thick arrow) and no definite intralesional vascularity

A pelvic USG [Figure 1] showed a complex 6.0 cm × 4.6 cm × 3.7 cm mass in the right adnexa, while the right ovary could not be separately identified, suggesting a possible ovarian origin. The mass had a cystic component with thick septae and also a solid component without definite evidence of posterior acoustic shadowing to suggest calcification. Minimal intralesional vascularity was identified. There was no free or loculated fluid in the pelvis. At this stage, our differential diagnosis included ovarian neoplasm, dermoid and tuboovarian abscess. Because of the absence of constitutional symptoms of infection, a tuboovarian abscess was considered relatively less likely. Further evaluation with MRI was recommended for characterization.

**Figure 2:**
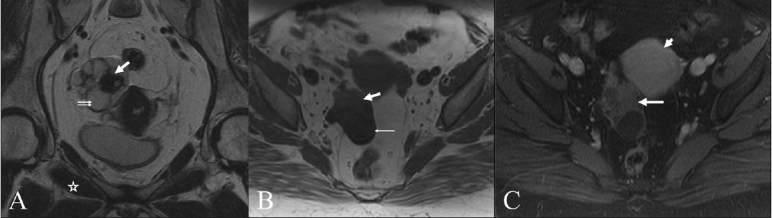
(A–C) Oblique coronal T2W (A), axial T1W (B) and axial postcontrast, fat-saturated T1W (C) MRI images show a complex right adnexal mass with cystic and solid components. The cystic component shows high-T2 signal (double arrow in A) and low-T1 signal (thin arrow in B), while the solid, fibrous component shows low-T2 signal (thick arrow in a) and intermediate T1 signal (thick arrow in B), isointense to skeletal muscle (star in A). Mild enhancement (long arrow in C) relative to the adjacent uterine myometrium (short arrow in C) is seen

MRI of the pelvis was performed on a 1.5-Tesla scanner, which showed a lobulated, complex, multiloculated right adnexal cystic mass [Figure 2] measuring 6.7 cm × 3.6 cm × 5.1 cm, with the right ovary not separately identified. There was a predominant cystic component demonstrating low-signal intensity on T1W and high-signal intensity on T2W images [Figure 2 A and B]. Solid components were also noted, which demonstrated intermediate signal intensity on T1W and low-signal intensity on T2W images – isointense to the skeletal muscle [Figure 2A and B]; there was mild postgadolinium enhancement [Figure 2C]. The features were suspicious for an ovarian cystic neoplasm with a fibrous component. There was no free fluid or adjacent pelvic lymphadenopathy.

The patient underwent total abdominal hysterectomy with bilateral salpingooophorectomy. The frozen section of the right ovarian mass demonstrated features of benign cystadenofibroma, a diagnosis that was confirmed on histopathology.

## Discussion

Ovarian cystadenofibroma is an uncommon benign neoplasm containing epithelial and fibrous stromal components, accounting for 1.7% of all benign ovarian tumors.[2] These tumors can be predominantly cystic, complex cystic with variable amounts of solid components or predominantly solid.[2,3] Because of their solid component or irregular thick septae, these masses are often diagnosed as malignant on preoperative imaging.

Even on gross examination at the time of surgery, a cystadenofibroma may resemble a malignant tumor. A frozen section diagnosis may be helpful in these cases because a correct diagnosis of cystadenofibroma in the operating room might save the patient from unnecessary extensive surgery.[2] Cystadenofibroma may occur in reproductive age group and an accurate preoperative diagnosis may help in avoiding extensive surgical procedure.[4]

On USG, a cystadenofibroma may show a solitary cyst or a multiloculated cystic mass, with solid nodules or papillary projections; 50% of the cases demonstrate increased vascularity.[6] USG cannot definitely characterize this tumor as its heterogenous appearance mimics a malignant ovarian neoplasm. A computed tomography (CT) scan also is of limited value in evaluating this tumor. In a study by Cho *et al*., all 16 cases of ovarian cystadenofibromas, presenting as complex cystic masses with solid components, were preoperatively misdiagnosed as malignant ovarian neoplasms on CT scan or MRI.[2]

MRI has been described as being the modality of choice for characterizing complex ovarian masses. Outwater *et al*.[5] first described the MRI feature of cystadenofibroma, reporting low-signal intensity (relative to the skeletal muscle) of the solid fibrous component of this tumor on T2W images. This finding has been further described in several other reports.[2–4,7] Other features that have been described include multiple tiny high-T2-signal intensity cysts associated with a low-signal intensity solid fibrous component, giving a sponge-like T2-imaging appearance[4] and multicystic foci with thickened septae, demonstrating low-T2-signal intensity corresponding to its fibrous nature.[3]

Other tumors with similar T2 characteristics due to a fibrous component are fibroma, fibrothecoma and Brenner tumor, which are all benign tumors. Ovarian fibromas, due to their solid component, mimic malignant lesions. However, because of excessive collagen and fibrous content, fibromas demonstrate low signal on T1W images and very low-signal intensity tissue on the T2W images.[7] Dense calcifications are also seen. These tumors may also undergo cystic degeneration, in which case there will be high-signal intensity on the T2W images.[7] Brenner tumor manifests as either a multilocular cystic mass with a solid component or as a small, predominantly solid mass. These tumors are composed of transitional cells, with a dense fibrous stroma, which results in low-signal intensity on T2W images. Extensive amorphous calcification is often seen within the solid component of these tumors.[8]

Malignant ovarian tumors with a fibrous component and low-T2-signal intensity are likely to be metastases from the gastrointestinal tract and struma ovarii. Metastases demonstrate a relatively low-T2 intensity of the fibrous component (although not as low as in a cystadenofibroma) and also show postgadolinium enhancement.[5] Struma ovarii has low-T2-signal intensity due to the viscid gelatinous material in the cysts, and there is no postcontrast enhancement.[5] The MRI features of a rare cystadenofibrocarcinoma have been described and the authors reported a predominant solid component with moderately high-T2-signal intensity and strong postgadolinium enhancement.[3]

This case illustrates the key MRI feature of this uncommon tumor; the presence of low-signal intensity on T2W images due to the fibrous tissue component. This feature may help in diagnosing similar cases in the future, when complex ovarian or adnexal lesions are seen on USG, and help avoid unnecessary surgery.

## References

[1] Czernobilsky B, Borenstein R, Lancet M (1974). Cystadenofibroma of the ovary. A clinicopathologic study of 34 cases and comparison with serous cystadenoma. Cancer.

[2] Cho SM, Byun JY, Rha SE, Jung SE, Park GS, Kim BK (2004). CT and MRI findings of cystadenofibromas of the ovary. Eur Radiol.

[3] Jung DC, Kim SH, Kim SH (2006). MR imaging findings of ovarian cystadenofibroma and cystadenocarcinofibroma: clues for differential diagnosis. Korean J Radiol.

[4] Takeuchi M, Matsuzaki K, Kusaka M, Shimazu H, Yoshida S, Nishitani H (2003). Ovarian cystadenofibromas: Characteristic magnetic resonance findings with pathologic correlation. J Comput Assist Tomogr.

[5] Outwater EK, Siegelman ES, Talerman A, Dunton C (1997). Ovarian fibromas and cystadenofibromas: MRI features of the fibrous component. J Magn Reson Imaging.

[6] Alcázar JL, Errasti T, Mínguez JA, Galán MJ, García-Manero M, Ceamanos C (2001). Sonographic features of ovarian cystadenofibromas: spectrum of findings. J Ultrasound Med.

[7] Jung SE, Lee JM, RHA SE, Byun JY, Jung JI, Hahn ST (2002). CT and MR Imaging of ovarian tumors with emphasis on differential diagnosis. RadioGraphics.

[8] Moon WJ, Koh BH, Kim SK, Kim YS, Rhim HC, Cho OK (2000). Brenner tumor of the ovary: CT and MR findings. J Comput Assist Tomogr.

